# Immunodominance in T cell responses elicited against different domains of detoxified pneumolysin PlyD1

**DOI:** 10.1371/journal.pone.0193650

**Published:** 2018-03-06

**Authors:** Els van Westen, Martien C. M. Poelen, Germie P. J. M. van den Dobbelsteen, Eliud O. Oloo, Martina M. Ochs, Nynke Y. Rots, Cecile A. C. M. van Els

**Affiliations:** 1 Centre for Infectious Disease Control, National Institute for Public Health and the Environment, Bilthoven, The Netherlands; 2 Sanofi Pasteur, Cambridge, MA, United States of America; 3 Sanofi Pasteur, Marcy L’Etoile, France; Instituto Butantan, BRAZIL

## Abstract

Detoxified pneumolysin, PlyD1, is a protein vaccine candidate that induces protection against infections with *Streptococcus pneumoniae* in mouse models. Despite extensive knowledge on antibody responses against PlyD1, limited information is available about PlyD1 induced T cell recognition. Here we interrogated epitope breadth and functional characteristics of the T cell response to PlyD1 in two mouse strains. BALB/c (H-2^d^) and C57BL/6 (H-2^b^) mice were vaccinated with Al(OH)_3_-adjuvanted or non-adjuvanted PlyD1, or placebo, on day 0, 21 and 42 and were sacrificed at day 56 for collection of sera and spleens. Vaccination with adjuvanted and non-adjuvanted PlyD1 induced anti-pneumolysin IgG antibodies with neutralizing capacity in both mouse strains. Adjuvantation of PlyD1 enhanced the serological responses in both strains. *In vitro* restimulation of splenocytes with PlyD1 and 18-mer synthetic peptides derived from pneumolysin revealed specific proliferative and cytokine responses. For both mouse strains, one immunodominant and three subdominant natural epitopes were identified. Overlap between H-2^d^ and H-2^b^ restricted T cell epitopes was limited, yet similarities were found between epitopes processed in mice and predicted to be immunogenic in humans. H-2^d^ restricted T cell epitopes were localized in pneumolysin domains 2 and 3, whereas H-2^b^ epitopes were scattered over the protein. Cytokine responses show mostly a Th2 profile, with low levels of Th1 cytokines, in both mouse strains. In conclusion, PlyD1 evokes T cell responses in mice directed against multiple epitope regions, that is dependent on Major Histocompatibility Complex (MHC) background. These results are important to understand human PlyD1 T cell immunogenicity, to guide cell mediated immunity studies in the context of vaccine development.

## Introduction

*Streptococcus pneumoniae* is a main cause of pneumonia, sepsis and meningitis in young children, which can be prevented by vaccination. Current pneumococcal conjugate vaccines (PCVs) protect against the serotypes present in the vaccine, but replacement of vaccine types with non-vaccine types in the general population is found [1]. Alternative pneumococcal vaccine candidates include conserved protein antigens that confer serotype-independent protection. Killed whole cell vaccines are an example of these alternative pneumococcal vaccines, and have been shown to induce a humoral and a Th17-type cell-mediated response in mice [2, 3]. This Th17-type response is thought to be important for protection against carriage, by promoting neutrophilic infiltration in nasopharyngeal mucosa [4, 5]. Serotype-independent coverage can be induced by conserved proteins, when they are used as a protein vaccine by itself or as a pneumococcal specific carrier protein in PCVs. Several conserved, mainly surface exposed, proteins are under investigation as vaccine candidates [6].

One of these protein vaccine candidates is pneumolysin (Ply), which has been identified in almost all clinical isolates [7], and has limited genetic variation across serotypes [8]. Ply is produced in the bacterial cytosol and released during bacterial lysis. Soluble Ply can form a pore cellular membranes which induces cell lysis, contributing to pneumococcal pathogenesis at various stages of the infection [9]. Ply promotes mucosal inflammation, increasing bacterial spread, and has been shown to be required for pneumococcal transmission in a mouse model [10]. Wall *et al* reported that persistently high levels of Ply in cerebrospinal fluid are associated with an increased risk of death in patients with pneumococcal meningitis [11]. Protection from an intranasal pneumococcal challenge in mice after immunization with Ply was already shown by Paton *et al* in 1983 [12]. However, Ply cannot be used as immunogen in its wild-type form because of its cytotoxicity [13]. Pneumolysoids are forms of Ply with reduced cytotoxicity, induced by site-directed mutagenesis or chemical detoxification. PdB, was the first pneumolysoids which had one mutation, W433F [14], and was shown to be protective in mouse models [15, 16], but retained some hemolytic activity [17, 18]. More recently, pneumolysoids ΔA146 and L460D were shown to confer protection in mouse models [19]. Structural design can facilitate engineering of pneumolysoids with reduced cytotoxicity but with maintained ability to elicit neutralizing antibodies [18].

Another pneumolysoid, PlyD1, with 3 point mutations (T65C, G293C, C428A) [18], was shown to protect against lethal pneumococcal challenges in mice by eliciting antibodies that can block cytolytic activity of native Ply *in vitro* and *in vivo* [20]. PlyD1, in combination with 2 other pneumococcal proteins (PcpA and PhtD), also protects against acute pneumococcal otitis media in an infant murinemodel [21]. In phase I clinical studies, PlyD1 was shown to be safe and to induce neutralizing antibodies in adults [22], and to be safe and immunogenic in adults, toddlers and infants when administered as part of a trivalent recombinant vaccine candidate, containing PcpA and PhtD as well [23]. Also an association was found between higher naturally acquired antibody levels against PlyD1 and 2 other proteins in the nasopharyngeal mucosa and a reduced risk for acute otitis media infection in infants [24]. Such human Ply specific antibodies are known to be capable of blocking pneumococcal colonization, as assessed in a mouse model [25]. These studies indicate that genetic detoxification of Ply is compatible with immunogenicity of the pneumolysoids and the induction of functional antibody responses to the wild-type protein.

While the immunogenicity of PlyD1 has been shown serologically, cell mediated immune recognition may be equally important. Pneumococcal protein-specific CD4 T helper cells have been implied in protection against pneumococcal infections [5, 26, 27]. CD4 T cells provide costimulatory signals to activated B cells, which are required for the induction of class switching of antibodies and development of plasma cells and memory B cells [28]. Moreover, CD4 T cells can contribute to the anti-bacterial response based on their functional cytokine differentiation. Three major CD4 T cell subsets can be classified, producing Th1 type cytokines IFN-γ, TNF-α and IL-2, Th2 type cytokines IL-4 and IL-5, or the Th17 type cytokine IL-17, respectively. Cytokines that have been specifically implied in protection against pneumococcal colonization and carriage are IFN- γ and IL-17 [4, 27, 29, 30]. Native Ply is known to evoke mixed Th1/Th17 responses in both mouse models and in humans [31, 32]. Yet, T cell immunogenicity of detoxified Ply has not been studied.

Therefore, in this study we aimed to unravel PlyD1 T cell immunogenicity in two mouse models with different genetic backgrounds after immunization with non-adjuvanted and adjuvanted PlyD1. Immunogenicity at the humoral level was confirmed. Furthermore, proliferative capacity, epitope specificity and breadth, and cytokine profiles of T cell responses were assessed in detail. Structural modeling was used to evaluate murine T cell-immunogenic domains of PlyD1 in relation to known antibody-targeted domains and predictions of human immunogenic T cell domains.

## Materials and methods

### Ethics statement

This study was approved by the Committee on animal Experimentation of the Antonie van Leeuwenhoek terrain (DEC-ALt, Bilthoven, the Netherlands) under permit number 201300139. Animal handling in this study was carried out in accordance with relevant Dutch national legislation, including the 1997 Dutch Act on Animal Experimentation. Before immunization, mice were anesthetized using isoflurane. Mice were sacrificed by cervical dislocation, while anesthetized using isoflurane.

### Antigens and peptides

Recombinant wild-type Ply and detoxified PlyD1 (amino acid changes T65C, G293C, C428A) were recombinantly expressed in Escherichia coli and purified by serial column chromatography and obtained from Sanofi Pasteur.

Overlapping synthetic peptides spanning full length Ply (SPD_1726 (Strain D39, serotype 2); 471 amino acids) [33] were produced in-house using F-moc chemistry, with a length of 18 amino acids (aa), and 12 aa overlap. Peptides including the mutations of PdB (W433F) and PlyD1 were produced. Using these peptides, thirty smart peptide pools, based on a 3-dimensional matrix, consisting of different combinations of 9 or 10 peptides were used to screen for candidate T cell immunogenic peptides.

### Mice and immunization

Female BALB/cOlaHsd (H-2^d^) mice (Harlan, Horst, The Netherlands) and C57BL/6J (H-2^b^) mice (Charles River, Erkrath, Germany) of 6–8 weeks old were divided into 4 groups. For each mouse strain per immunization series, groups were subcutaneously immunized at day 0, 21 and 42 with 200 μl vaccine containing either 10 μg PlyD1 (Sanofi Pasteur) (n = 5/group) or as placebo only with Phosphate Buffered Saline (PBS, Gibco Waltham, USA) (n = 3/group), formulated with or without 0.56 mg/ml aluminum hydroxide (Al(OH)_3_) (2% Alhydrogel®, Invivogen, Toulose, France), respectively. Blood samples were taken by orbital puncture at day 0, 21 and 42. Animals were sacrificed at day 56 and blood and spleens were collected. For each mouse strain, results were obtained from two independent immunization series showing identical patterns. Results shown are from one study. In one of the immunization series one BALB/c mouse in the PlyD1 vaccinated group died.

### Antibody levels

Ply-specific antibodies were determined in sera using ELISA. Plates were coated overnight at room temperature with 5 μg/ml Ply in PBS (Gibco). Plates were washed using 0.03% Tween-80 (Merck, Amsterdam, The Netherlands) in tap water (wash buffer). Sera were serially diluted (150 μl) in plates in 0.1% Tween-80 in PBS and incubated for 2 hours at 37°C. Unbound antibodies were washed away three times using wash buffer and goat-anti-mouse HRP-labeled conjugates, IgG, IgG1, IgG2a or IgG2c (Southern Biotech, Birmingham, USA) diluted in 0.5% Protifar (Nutricia), 0.1% Tween-80 in PBS were added to the plates and incubated for 2 hours at 37°C. Then, free conjugate was removed by washing three times using wash buffer, and TMB-substrate (Sigma-Aldrich, Zwijndrecht, The Netherlands) was added. After 10 minutes, the reaction was stopped by addition of H_2_SO_4_ (Boom, Meppel, The Netherlands). Specific antibody levels were determined by measuring the OD at 450 nm and calculating titers, using the computer program Gen5, as the dilution at 50% of the maximum OD. For the purpose of data analyses, samples below the detection limit of a titer of 150 were set to a titer of 50. Geometric means with standard deviation (SD) were calculated per group. Ratios of IgG2a/IgG1 (for BALB/c mice) and IgG2c/IgG1 (for C57BL/6 mice) were calculated for each individual mouse and the average per group was determined.

### Pneumolysin-neutralizing capacity

The capacity of sera to neutralize the cytotoxic activity of Ply was determined using a hemolysis inhibition micro assay (HIMA). Cholesterol was removed from the sera using Dextran-sulphate (Sigma-Aldrich), to prevent Ply from binding. Samples were serially diluted in 0.5% Bovine Serum Albumin (BSA, Sigma-Aldrich) in Hank’s Balanced Salt Solution (Gibco) in a 96-well V-bottom plate and 1 ng Ply was added and incubated for 30 minutes at 37°C with shaking. Fresh sheep red blood cells (Biotrading, Mijdrecht, The Netherlands) were washed and diluted to 3% in PBS and 50 μl was added to the plates. After 30 minutes incubation at 37°C with shaking, plates were centrifuged and supernatants were transferred to a 96-well flat-bottom plate. Inhibition of lysis of the erythrocytes was measured at 450 nm and calculated, using Gen5, as the dilution at 50% of the maximum OD and expressed as geometric means with SD per group.

### Cell isolation and stimulation

Splenocytes were homogenized and used either freshly or after storage in 10% Dimethyl sulfoxide (Sigma-Aldrich) at -135°C. Splenocytes were tested for each individual mouse. Cells were stimulated in Iscove’s Modified Dulbecco’s Medium (Invitrogen) containing 10% heat-inactivated Fetal Bovine Serum (HyClone, Logan, USA) and penicillin/streptomycin/glutamine (Invitrogen), in 96-well U-bottom plates at a concentration of 1.5 x 10^5^ or 1.0 x 10^5^ cells/well. Whole antigen (PlyD1, 1 μg/ml), 3-dimensionally-pooled peptide pools (1 μM per peptide), or medium control were used for identification of candidate epitopes on freshly-isolated cells. Epitope confirmation and cytokine analysis were performed by stimulating with whole antigen (PlyD1, 1 μg/ml), single synthetic peptides (1 μM) or medium only (negative control) on freshly isolated cells (BALB/c mice) or on thawed cells (C57BL/6 mice). Stimulations were performed in quadruplicate wells and incubated for 5 days at 37°C with 5% CO_2_. Thereafter, 100 μl supernatant from each well was harvested and stored at -20°C until cytokine measurement, and cell cultures were processed for proliferation analysis.

### Proliferation

To determine cell proliferation in the stimulated cultures, at day 5 tritium thymidine (18 kBq/well) was added to the 96-well plates for overnight incubation at 37°C with 5% CO_2_, to be incorporated in the cellular DNA with every cell division. Cells were harvested on a filter (Filtermat A, Perkin Elmer, Oss, The Netherlands) and incorporated label was determined as counts per minute (cpm) using a MicroBeta Counter (Perkin Elmer). Mean cpm from quadruplicate wells were used to calculate the Stimulation Indices (SI) by dividing the mean cpm of stimulated cultures by the mean medium control. Results are shown as mean SIs per group with standard deviations. SIs >2 were considered positive and in single peptide stimulated cultures indicative for the identification of an epitope. Immunodominance of epitopes was determined based on ranking of SIs in response to single synthetic peptides per mouse strain. The shared sequence between overlapping peptides with SIs >2 were considered to represent the epitope core.

### Cytokine responses

Cytokine production was measured in pooled cell culture supernatants from quadruplicate wells, using a multiplex immunoassay (MIA). Concentrations of cytokines IL-2, IL-4, IL-5, IL-10, IL-17, TNF-α, and IFN-γ were determined using a mouse cytokine kit (Millipore, Amsterdam, The Netherlands) according to instructions of the manufacturer. Samples were measured and data were analyzed with Bio-Plex200 and Bio-Plex Manager Software 5.0 (Bio-Rad Laboratories). Results are shown as geometric mean concentration per group in pg/ml.

### In silico prediction of human Ply T cell immunogenic epitopes

The EpiMatrix algorithm (EpiVax Inc.) was used to evaluate the probability of Ply derived peptide binding to a panel of eight common HLA-DRB1 alleles (DRB1*01:01, DRB1*03:01, DRB1*04:01, DRB1*07:01, DRB1*08:01, DRB1*11:01, DRB1*13:01, and DRB1*15:01), covering over 98% of the human population to predict human T cell immunogenicity of Ply [34]. An EpiMatrix hit indicates any HLA-DRB1 allele recognizing a frame of 9 amino acids. EpiBars were identified when at least 4 alleles were predicted to recognize a frame of 9 amino acids. The level of homology of Ply and derived EpiBars to the human proteome was analyzed by determining the Human Janus Homology Score [35]. Analysis was based on the full protein sequence of Ply (SP_1923, *Streptococcus pneumoniae* TIGR4).

### 3D modelling of T cell epitopes

Crystal structures of wild-type Ply have been published [36–38]. A three-dimensional structural model of PlyD1 was computationally constructedusing a well-resolved and complex x-ray crystal structure of wild-type pneumolysin (PDB ID: 5AOD) as the template [37]. The structural coordinates of the template were obtained from the Protein Data Bank [39]. PlyD1 mutations T65C, G293C and C428A were introduced into the wild-type 3D structure using BioLuminate (Release 2015–3, Schrödinger, LLC, New York, NY, 2015). For each substitution, all residues within a 4 Å cut-off distance from the mutation site were refined in order to accommodate the newly introduced amino acid. Solvent effects were accounted for using an implicit solvation model. Mapping of T cell epitopes and generation of images was performed using PyMol Molecular Graphics software (Version 1.7.4, Schrödinger, Inc).

### Statistical analyses

For statistical analyses, all serology data, stimulation indices and cytokine results were log-transformed and differences between groups were determined using a T test. Differences in cytokine results between antigen-stimulated cultures and medium controls were determined using a T test. P-values <0.05 were considered significant. Analyses were performed in SPSS19.0 and GraphPad Prism6.

## Results

### IgG responses induced by PlyD1 are predominantly IgG1 and have neutralizing capacity

**Fig 1:**
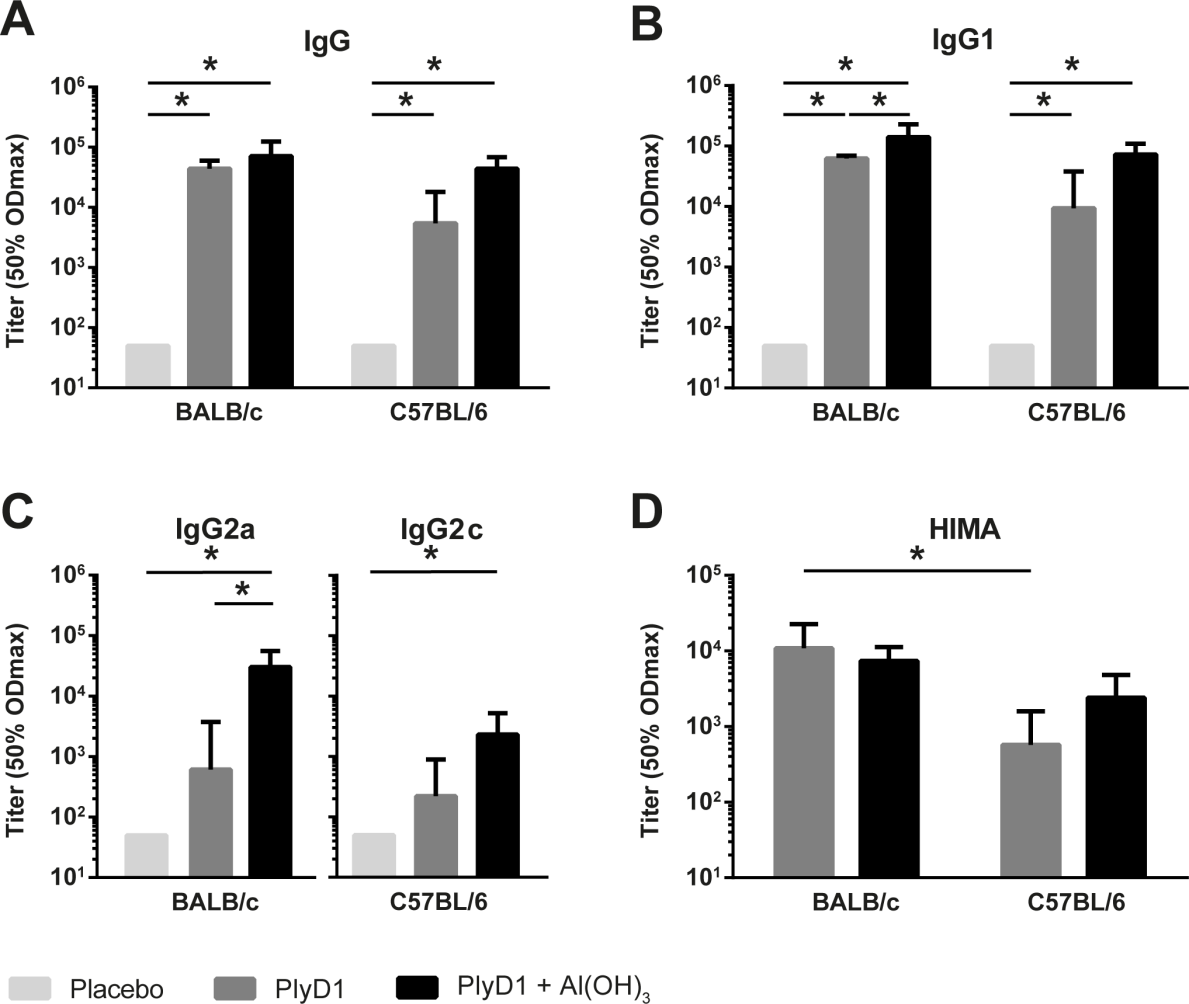
Serological responses after 3 immunizations with PlyD1. BALB/c and C57BL/6 mice were immunized 3 times with PlyD1, with or without aluminum hydroxide, or with PBS placebo. Ply-specific antibody titers are shown for total IgG (A), IgG1 (B), IgG2a for BALB/c mice and IgG2c for C57BL/6 mice, respectively (C). Neutralizing capacity of antibodies against hemolytic activity of Ply was determined using HIMA (D). Geometric means of (reciprocal) titers at 50% ODmax with standard deviations are displayed per group.

Non-adjuvanted PlyD1 induced high levels of total IgG antibodies directed against Ply in both BALB/c and C57BL/6 mice. Specific IgG levels were enhanced after addition of aluminum hydroxide, but differences were not significant (Fig 1A). Patterns of IgG1 antibodies were similar to those found for total IgG, except that addition of an adjuvant significantly increased IgG1 levels in BALB/c mice (Fig 1B). For IgG2a and IgG2c, in BALB/c and C57BL/6 mice respectively, similar patterns were found compared to total IgG and IgG1, though at a lower level (Fig 1C). Levels of IgG2a, in BALB/c mice, were significantly higher after vaccination with PlyD1 with adjuvant compared to PlyD1 alone. Immunization with PlyD1 induced ratios of IgG2a/IgG1 in BALB/c mice of 0.01 and IgG2c/IgG1 ratios for C57BL/6 mice of 0.02. These ratios were below 1, indicating that PlyD1 alone induces a Th2 dominated immune response. When Al(OH)_3_ was added to PlyD1, ratios of IgG2a/IgG1 and IgG2c/IgG1 increased to 0.22 and 0.03 for BALB/c and C57BL/6, respectively.

Assessment of sera in the hemolysis inhibition assay showed that antibodies induced by PlyD1 have neutralizing capacity in PlyD1 vaccine groups immunized with and without adjuvant in both mouse strains (Fig 1D). In BALB/c mice, vaccination with non-adjuvanted PlyD1 induced significantly higher neutralizing antibody titers than in C57BL/6 mice.

### Identification of murine T cell epitopes of PlyD1

**Fig 2:**
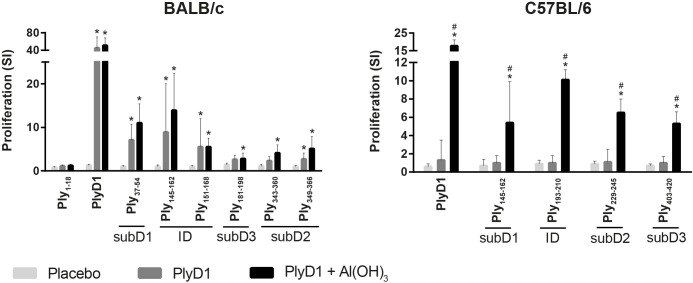
Determination of ID and subD PlyD1 T cell epitopes. Splenocytes from mice immunized with PlyD1, PlyD1+Al(OH)_3_ or PBS placebo were tested, freshly in BALB/c mice and after thawing in C57BL/6 mice, against PlyD1 and individual peptides from positively tested smart pools. ID regions are peptides with highest proliferation, subD regions are epitopes with subsequent proliferative levels. Proliferation is expressed as mean SI with standard deviation per group. * p value < 0.05 compared to the placebo group. # p value < 0.05 comparing PlyD1 with and without Al(OH)_3_.

Splenocytes from PlyD1 immunized BALB/c and C57BL/6 mice proliferated to PlyD1 and to certain smart peptide pools. Specific immunogenic epitopes were identified by confirmation proliferation using single peptides. One immunodominant (ID) and three subdominant (subD) T cell-immunogenic regions were identified for each mouse strain (Fig 2, Table 1). In the BALB/c model, individual peptide Ply_145-162_ induced the strongest T cell proliferation. The partially overlapping peptide Ply_151-168_ also induced proliferation, indicating that the ID T cell epitope for BALB/c mice (H-2^d^) has its core in the overlapping sequence of these peptides. Single peptides that induced a subD T cell response in splenocytes of BALB/c mice were Ply_37-54_, Ply_181-198_ and Ply_343-360_/Ply_349-366_. In C57BL/6 mice (H-2^b^) peptide Ply_193-210_ was the ID epitope, with peptides Ply_145-162_, Ply_229-246_ and Ply_403-420_ being subD. Only for synthetic peptide Ply_145-162_ T cell proliferation was found in splenocytes of both mouse strains. None of the recognized epitopes, in either mouse strain, contained any of the Ply aa positions being mutagenized in PdB or PlyD1. T cell proliferation after stimulation with subD peptide Ply_43-59_ was higher using splenocytes from BALB/c mice after vaccination with adjuvanted PlyD1, compared to PlyD1 alone (Fig 2). In C57BL/6 mice, proliferation to single peptides could only be detected after vaccination with PlyD1 adjuvant with aluminum hydroxide. In all experiments, stimulation with the whole protein PlyD1 induced the highest proliferative responses when compared to stimulation with peptide pools or single peptides representing regions of PlyD1, in both mouse strains and for both cell types.

**Table 1:** Identified immunodominant and subdominant T cell regions in PlyD1.

	Peptide^1^	AA	Sequence	
**BALB/c**	Ply_145-162_/Ply_151-168_^2^	151–162	EKITAHSMEQLK	
	Ply_37-54_	37–54	NQLPDEFVVIERKKRSLS	
	Ply_181-198_	181–198	NSVHSGEKQIQIVNFKQI	
	Ply_343-360_/Ply_349-366_^2^	349–360	DYVETKVTAYRN	
**C57BL/6**	Ply_193-210_	193–210	VNFKQIYYTVSVDAVKNP	
	Ply_145-162_	145–162	PARMQYEKITAHSMEQLK	
	Ply_229-246_	229–246	SAERPLVYISSVAYGRQV	
	Ply_403-420_	403–420	DLTAHFTTSIPLKGNVRN	

^1^ Immunodominant epitopes are underlined.

^2^ For overlapping stimulatory peptides aa positions and sequences are given for shared amino acids.

### Immunization with PlyD1 skews immune responses towards a Th2 cytokine response

**Fig 3:**
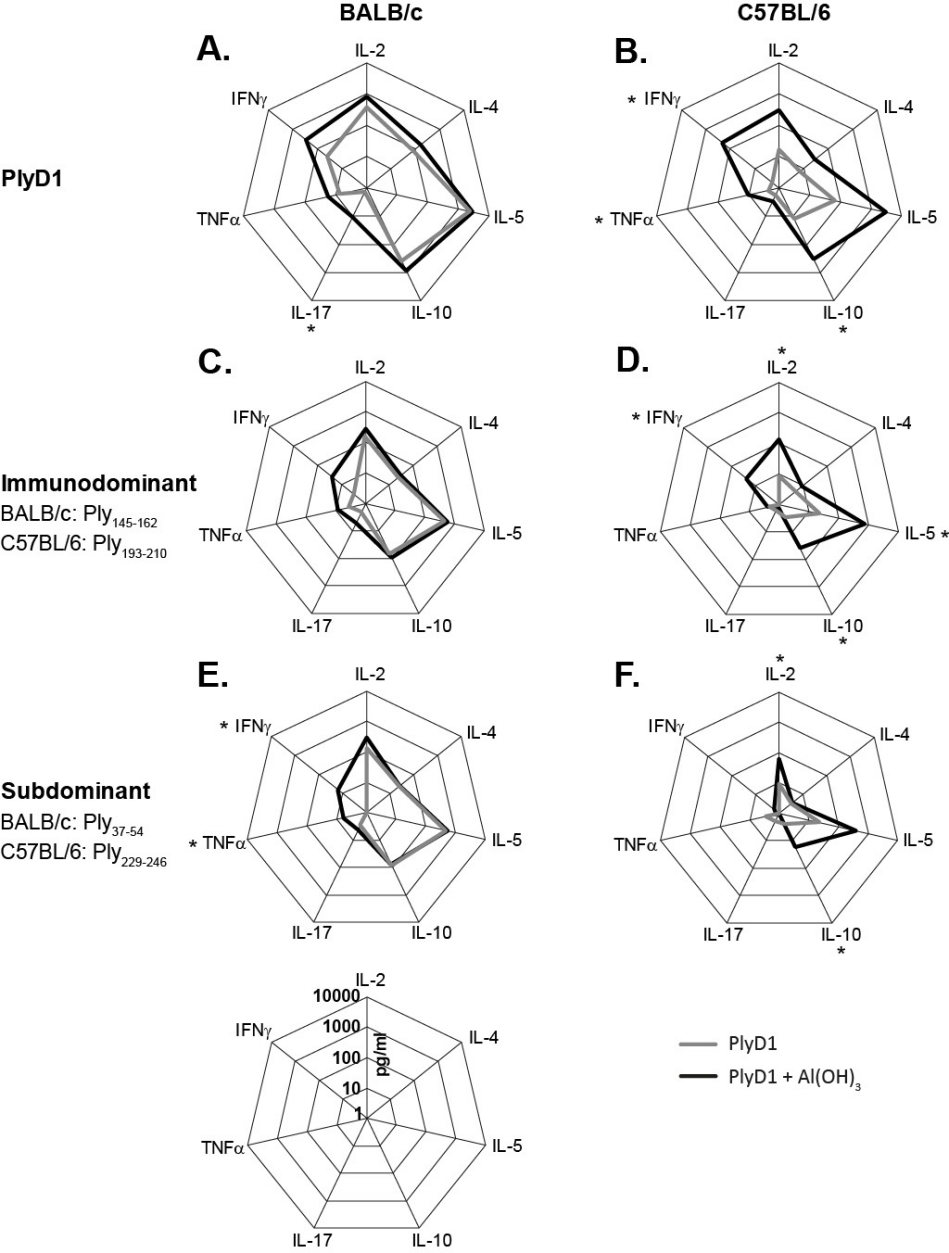
Cytokine profiles in response to PlyD1 and peptides. Geometric mean cytokine levels of groups of BALB/c (A, C, E) or C57BL/6 mice (B, D, F), vaccinated with PlyD1 in the absence (gray lines) or presence (black lines) of adjuvant, determined in supernatants of splenocytes after 5 days of *in vitro* stimulation with PlyD1 (A, B), the ID peptide (C, D), or a subD peptide (E, F) from Ply. Geometric mean cytokine levels are shown in log10 scale. For both mouse strains, a representative subD epitope is depicted. Significant differences between responses induced by PlyD1 alone or PlyD1 adjuvanted with aluminum hydroxide are marked with an *.

Levels of Th1-, Th2- and Th17-type cytokines were determined in culture supernatants after five days *in vitro* restimulation of splenocytes with PlyD1 or peptides representing ID or subD epitopes (Fig 3, S1 Table). In BALB/c mice, whole PlyD1 induced cytokine profiles were similar for both vaccinated groups, irrespective of the presence of adjuvant, except that IL-17 was induced and the level of IFN-γ enhanced after administration of the adjuvanted vaccine (Fig 3A). In these supernatants, relatively high geometric mean concentrations were found for IL-5, IL-10, IL-2 and IFN-γ, with low levels of IL-4, TNF-α and IL-17. In splenocytes from C57BL/6 mice vaccinated with PlyD1 alone lower levels of cytokines were detected *in vitro*, of which IL-10, IFN-γ and TNF-α were significantly enhanced after vaccination with adjuvanted PlyD1 (Fig 3B). These data confirm at the cytokine level that in both mouse strains adjuvantation of PlyD1 with aluminum hydroxide diminishes Th2 cytokine dominance of the PlyD1 immune response.

Levels of cytokines after *in vitro* restimulation with single ID or subD peptides were lower compared to those after restimulation with whole protein. Yet, the cytokine profiles in response to ID and subD peptides followed PlyD1 restimulated patterns in both BALB/c and C57BL/6 mice (Fig 3C–3F).

### Mapping of T cell epitopes in different domains of pneumolysin

**Fig 4:**
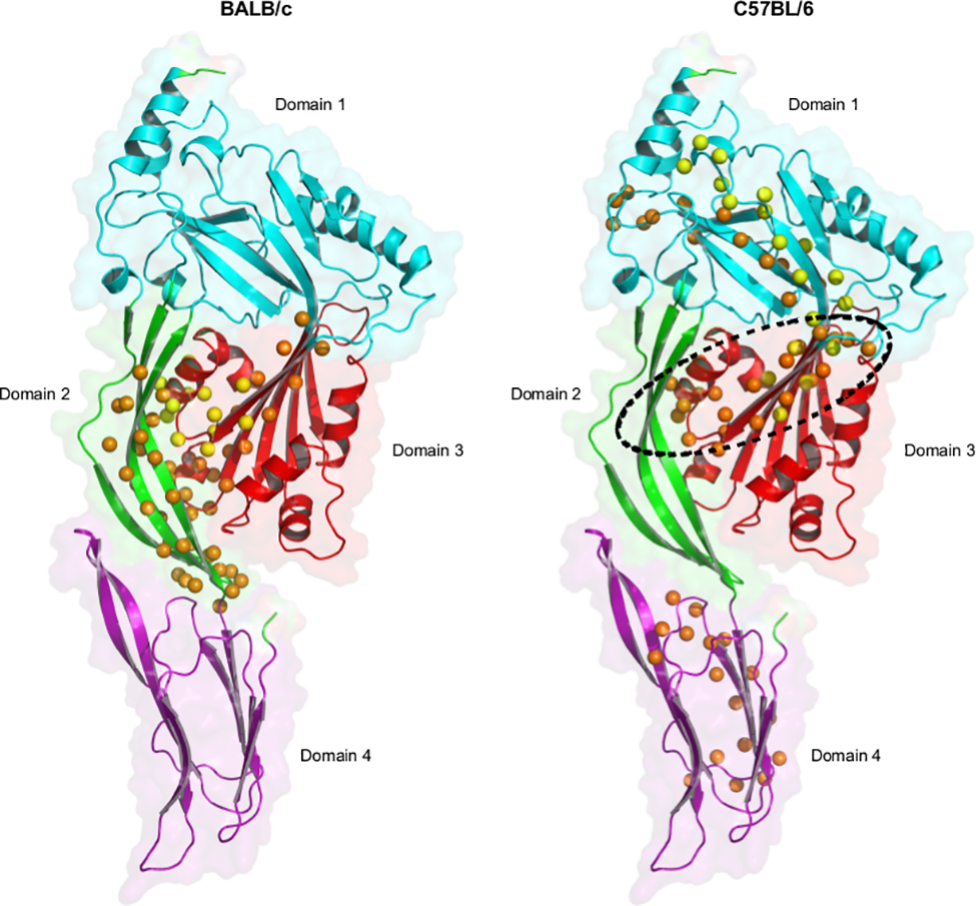
3D modelling of T cell epitopes in Ply for BALB/c and C57BL/6 mice. Yellow spheres represent the C-ß atoms of the ID epitope and orange spheres represent the C-ß atoms of the subD epitopes. The dotted circle represents the overlap region between both mouse strains. Colors of the protein structure display the different domains of Ply: cyan indicates domain 1 (amino acids 1–21, 58–147,198–243,319–342), green indicates domain 2 (amino acids 22–57,343–359), red indicates domain 3 (amino acids 148–197,244–318), and purple indicates domain 4 (amino acids 360–469).

ID and subD epitopes in both BALB/c (H-2^d^) and C57BL/6 (H-2^b^) mice (Table 1) were mapped onto a 3D structural model of Ply (Fig 4). Epitopes identified in both mouse strains are predominantly located in buried β-sheet regions of the protein, which are the most hydrophobic.

For BALB/c mice, the ID epitope is located in domain 3 and the subdominant epitopes resided mainly in domain 2. For C57BL/6 mice, the ID epitope is mostly located in domain 1 with a small part in domain 3, while subD epitopes of C57BL/6 mice are scattered over the protein but do not locate in domain 2. One immunogenic T cell region in domain 3, Ply_151-162_, clearly overlaps between mouse strains, being the core of the ID epitope of BALB/c mice and part of a subD epitope in C57BL/6 mice. Another short sequence Ply_193-198_, was shared by the ID epitope in C57BL/6 mice and a subD epitope in BALB/c mice. In the structural model, these shared T cell sequences consist of two β-strands and a stretch of α-helical fold within domain 3.

### Overlap between mouse T cell immunogenic PlyD1 regions and prediction of human immunogenic regions

**Fig 5:**
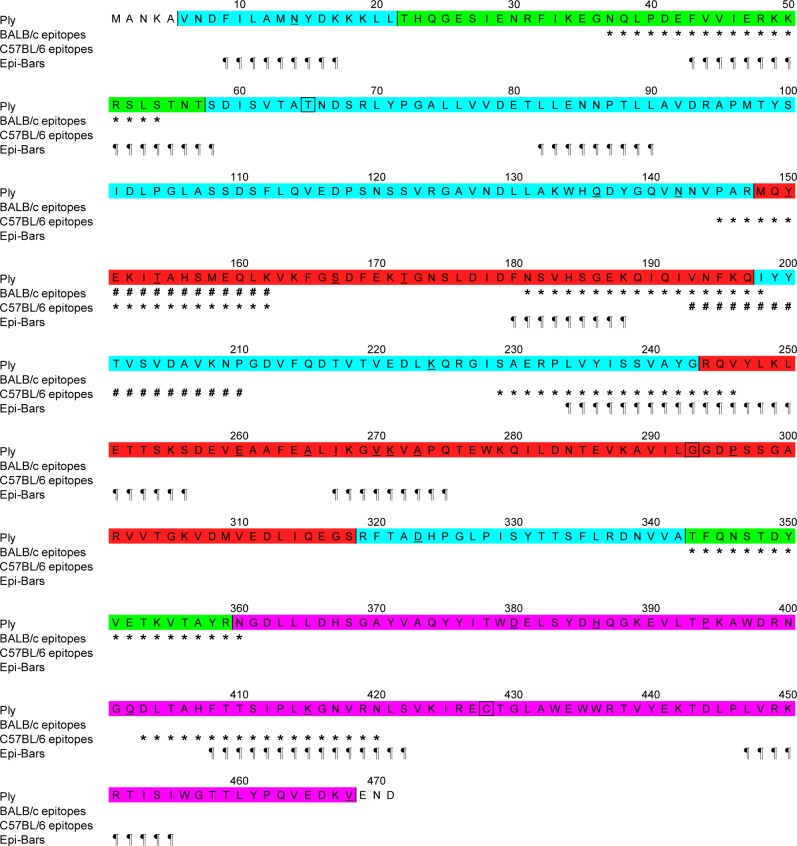
Ply sequence with localization of BALB/c and C57BL/6 T cell-immunogenic regions and predicted HLA-DR binding regions. Immunodominant epitopes of both BALB/c and C57BL/6 mice are indicated with a #, and subdominant epitopes with a *. ¶ shows the Epi-bars that are *in silico* predicted for HLA-DR-binding epitopes in humans. Mutated aa residues of PlyD1 are squared and positions with allelic variation as described by Jeffries *et al* [8] are underlined. Colors, cyan, green, red and purple, indicate domains 1, 2, 3, and 4 of Ply, respectively.

*In silico* screening of the Ply protein sequence to predict human immunogenic regions based on the probability of binding to a set of common HLA-DR alleles [34] resulted in 188 EpiMatrix Hits. Corrected for the length of the protein an overall EpiMatrix score of -9.40 was found. This indicated an average potential for T cell immunogenicity, as can be expected in a randomly generated protein sequence. The Janus Protein score was 0.80, predicting limited cross-reactivity with human peptide sequences. Further analyses showed 9 Epibars, immunogenic regions of the protein, of which two were partially overlapping (Fig 5). These Epibars were reactive for the majority of the eight selected HLA-DRB1 types, representing over 98% of the human population. Two of the identified human Epibars overlap with subD epitopes in BALB/c mice (H-2^d^), Ply_43-54_ and Ply_181-188_, and two other Epibars overlap with subD epitopes of C57BL/6 mice (H-2^b^), Ply_234-246_ and Ply_408-420_. No overlap was found between human Epibars and the identified ID epitopes of both mouse strains.

## Discussion

In this study, immunogenicity of PlyD1 at the humoral level was confirmed in two mouse strains. More importantly, new insights were gained into PlyD1’s cellular immunogenicity and its immunodominant T cell epitope regions, knowledge which can be used in the context of vaccine development and for the study of Ply-specific T cell immunity.

PlyD1 alone was capable of inducing comparable IgG levels in BALB/c and C57BL/6 mice. Yet, at the T cell level PlyD1 seemed less immunogenic in C57BL/6 mice since they required an adjuvanted PlyD1 vaccine for the induction of detectable *in vitro* anti-PlyD1 T cell proliferation. Neutralizing capacity of antibodies against Ply was significantly lower in C57BL/6 mice compared to BALB/c mice after PlyD1 immunization without adjuvant, perhaps related to a more limited T cell response in this C57BL/6 mouse group. BALB/c mice are more Th2-prone [40], which might explain the reduced need for an adjuvant and the higher neutralizing capacity achieved in this mouse strain. Small increments in IgG ratio after adjuvantation, albeit more distinct in BALB/c mice than in C57BL/6 mice, may suggest that aluminum hydroxide reduces the Th2 profile of the PlyD1 immune response in both mouse strains. Overall, addition of the aluminum hydroxide adjuvant to PlyD1 seemed to enhance its immunogenicity at both the antibody and T cell level.

Our data is showing that PlyD1 induces IgG titers and neutralizing antibodies in both BALB/c and C57BL/6 mice corroborate with previous preclinical observations of serological immunogenicity [20, 41, 42]. In our study, immunization of both mouse strains with PlyD1 resulted in higher levels of IgG1 than of other IgG subclasses, as was also shown by Verhoeven *et al* for C57BL/6 mice [43]. IgG1 is mainly propagated by IL-4, which is produced by Th2 cells [44]. The cytokine response to PlyD1 indeed seemed dominated by the Th2 cytokine IL-5, with intermediate levels of IL-4, Th1 cytokines, and lower levels of IL-17A detected after *in vitro* splenocyte culture. The use of thawed splenocytes for C57/BL6 mice in the cytokine analysis might have affected their in vitro responsiveness yet overall both mouse strains showed a similar cytokine dominance pattern. In recent studies, the importance of Th17 cells in protection against infections with pneumococci was highlighted [4, 5, 29, 31, 45]. Basal IL-17A responsiveness to PlyD1 was found in our mouse study, as opposed to strong Th17 responses promoted by natural Ply. This likely relates to the lack of hemolytic activity, which is responsible for stimulating the inflammasome [32, 46]. In our study, levels of PlyD1-induced IFN-γ and IL-17A responses were relatively more enhanced by aluminum adjuvantation than Th2 cytokines IL-4 and IL-5, indicating that adjuvants may not only promote PlyD1 immunogenicity but also influence functional T cell differentiation. Other adjuvants or administration routes may contribute to the optimization of the T helper cell response to PlyD1, but this needs further investigation. Levels of cytokines shown in our study should be interpreted or compared with care, since harvesting of culture supernatants at day 5 might not be equally optimal for all cytokines.

Molecular hallmarks of Ply presentation to CD4 T cells have so far remained elusive. It has been shown that natural Ply triggers *in vitro* human innate responses and polyclonal CD4 T cell activation, ahemolytic Ply to a lesser degree than hemolytic Ply [47]. We elucidated PlyD1’s T cell immunogenicity at the level of the recognized epitope regions. CD4 T cell epitopes are protein fragments generated by the processing machinery of antigen presenting cells and presented by MHC class II molecules at the cell surface. In each mouse strain studied, we identified 1 ID and 3 subD Ply specific T cell epitopes using splenocytes after PlyD1 immunization. There was limited overlap between epitope regions in the different mouse strains, but subD epitopes identified in each mouse model showed overlap with predictions for human immunogenic regions. None of the identified T cell epitopes overlapped with aa residues mutated in PlyD1[20] or in other pneumolysoids such as PdB, ΔA146 and L460D [14, 18, 19], which suggests that these mutations, made to reduce cytotoxicity, should not affect recognition of epitopes processed from native Ply by vaccination-induced T cells. T cell epitopes for BALB/c mice were mainly located in Ply domains 2 (Ply_37-54_ and Ply_349-360_) and 3 (Ply_151-162_ and Ply_181-198_), whereas in C57BL/6 mice they were present in domains 1 (Ply_193-210_ and Ply_229-246_), 3 (Ply_145-162_), and 4 (Ply_403-420_), but not domain 2. Only Ply_145-162_ was targeted in both mouse strains. This region is sandwiches at the immediate interface between domains 1 and 3, a part of the protein that undergoes dramatic refolding during pore-formation [37]. The importance of domain 4, which is not significantly refolding, for human T cell responses is addressed by Gray *et al* [31]. Notably, all identified murine T cell epitopes, irrespective of the particular domain involved, co-locate closely to secondary β-strand structures in Ply [37]. These hydrophobic structures likely contain MHC class II binding motifs, n a MHC allele-dependent manner [48]. The selective overlap between Ply-specific T cell epitopes found for BALB/c and C57BL/6 mice confirms the different binding motifs of their MHC haplotypes, H-2^d^ and H-2^b^, respectively. Importantly, the combined mouse models could bridge *in vivo* PlyD1 T cell immunogenicity data to *in silico* Ply epitope predictions in humans. The predicted human epitope panel had considerable overlap with the epitopes proven to be *in vivo* processed and presented in mice (Fig 5).

Ply is highly conserved, yet a few differences in alleles of this pore-forming antigen have been identified in a limited number of carriage strains and invasive isolates [8, 49, 50]. Two overlapping immunogenic peptides, Ply_145-162_ and Ply_151-168_, spanned aa positions for which natural variants have been identified (Y150H, Y150M or S167F), as described by Jefferies *et al* [8]. However, the shared core sequence (Table 1) between the 2 peptides is not affected. Another targeted peptide, Ply_403-420_, has been associated with a 7 aa insertion at aa 415 [8]. Whether such natural variations may affect the recognition of Ply alleles by Ply-specific T cells in humans will need further investigation.

The Ply specific T cell epitope mapping yielded another noteworthy observation. Although linkage of B- and T cell responses to the same antigen is important for cognate T cell help to B cells, their targeted epitopes do not necessarily have to overlap. This seemed indeed the case for Ply, as BALB/c T cell epitopes were identified in domains 2 and 3, while different BALB/c-derived monoclonal antibodies to Ply recognize domain 1 (n = 1 clone) or domain 4 (n = 3 clones) [51]. Therefore, from a vaccine development perspective, if both T- and B cell responses to Ply are desired all domains may be needed.

In summary, PlyD1 is B cell-immunogenic in BALB/c and C57BL/6 mice and induces antibodies with neutralizing capacity. More importantly, various ID and subD PlyD1 T cell epitopes were elucidated, which varied between the two tested mouse strains. Recognition of such T cell epitopes may drive cytokine responses and help B cell responses protective against pneumococcal infections. Together, these epitopes identified in mice could be used as a lead for human T cell studies, since PlyD1 aa sequences selected *in vivo* by mouse models had considerable overlap with human *in silico* predicted epitopes. If identified PlyD1 specific T cell regions can also be confirmed in humans, they could possibly be used either as a pneumococcus-specific carrier protein in a polysaccharide conjugate vaccine or as a component of a protein vaccine, requiring further investigation.

## Supporting information

S1 Table Statistical significance of differences between cytokine levels in supernatants of antigen stimulated splenocytes versus medium controls. (XLSX)
